# Integrating CEN ISO/TS 82304-2 in the Catalan Health App Assessment Framework: Comparative Case Study

**DOI:** 10.2196/67858

**Published:** 2025-06-04

**Authors:** Berta Llebot Casajuana, Petra Hoogendoorn, Maria Villalobos-Quesada, Carme Pratdepàdua Bufill

**Affiliations:** 1 Fundació TIC Salut Social, Ministry of Health, Government of Catalonia, Aragó 330-332, Barcelona, 08009, Spain, 34 667162080; 2 National eHealth Living Lab, Public Health and Primary Care Department, Leiden University Medical Centre, Albinusdreef 2, Leiden, 2333 ZA, The Netherlands

**Keywords:** mHealth, mobile health, mobile applications, assessment, criteria, quality, regulatory, comparative analysis, health app, comparative case study, Catalonia, health apps assessment, ISO, certification, CEN ISO/TS 82304-2, mixed methods, framework, TIC Salut Social Foundation

## Abstract

**Background:**

Health apps are increasingly being used to promote health, manage diseases, and deliver health care services. Still, there is scarce objective information regarding their quality beyond the required Conformité Européenne mark for medical apps, leading to potential risks for users. To address these challenges, several authorities have developed health app assessment frameworks. In 2017, the TIC Salut Social Foundation (FTSS) in Catalonia developed its own health app assessment framework, which has been in use since that year. The publication of CEN ISO/TS 82304‐2 (abbreviated as 82304‐2)—a Technical Specification for assessing health apps—and the cocreation of the Label2Enable 82304‐2 handbook for certified assessment organizations provide a unique opportunity to harmonize app assessments across the European Union.

**Objective:**

This study aimed to perform a comparative analysis of the FTSS assessment framework with 82304‐2 to explore the integration of 82304‐2 in Catalonia. Our broader aim was to provide this methodology for health authorities elsewhere to consider integrating 82304‐2 or other evaluation frameworks.

**Methods:**

For the comparative analysis, a mixed methods approach was used, combining a qualitative case study with a quantitative analysis of the 2 frameworks. The qualitative evaluation covered rationale for assessment, framework characteristics, governance, workflows, quality aspects, and quality requirements. For the quantitative analysis, all FTSS and 82304‐2 requirements were translated into concepts and subconcepts. A scoring system identified matches of the frameworks with these subconcepts, with scores ranging from 0 (no match) to 0.5 (partial match) and 1 (full match). Integration was evaluated considering several scenarios, including adopting the Label2Enable 82304‐2 handbook, adopting the 82304‐2 requirements, adapting the 82304‐2 requirements to local needs, and maintaining the current FTSS framework.

**Results:**

The main difference between the frameworks was the app usage–based assessment (FTSS) versus evidence- and app usage–based assessment (82304‐2). All 120 FTSS requirements and 74 quality aspect–related 82304‐2 requirements were translated into 78 concepts and 97 subconcepts. Overall, 48% (47/97) of the subconcepts were found in both frameworks, 39% (37.5/97) were specific to 82304‐2, and 13% (12.5/97) were specific to FTSS. All 82304‐2-specific subconcepts and thus all 82304‐2 requirements were found to be relevant to FTSS. FTSS decided to integrate (adopt and adapt) all 74 82304‐2 requirements. In total, 5 FTSS-specific requirements were included in the Label2Enable 82304‐2 handbook, while another 4 rigor-enhancing requirements, 1 scope-expanding requirement, and 1 context-specific requirement would be assessed on top.

**Conclusions:**

The comprehensive comparative analysis of the FTSS framework and 82304‐2 enabled FTSS decision-making to integrate all 82304‐2 quality requirements and adopt the Label2Enable 82304‐2 handbook in the future. The many new and all relevant 82304‐2 concepts, the rigor of the handbook, and the few remaining FTSS-specific requirements are expected to be indicative of 82304‐2’s potential to make harmonized, robust health app assessments common in Catalonia and elsewhere. FTSS encourages other authorities to perform a similar evaluation.

## Introduction

### Background

Health apps refer to apps designed for managing, maintaining, or improving the health and well-being of individual persons or the delivery of care, and thus include both medical and wellness apps [1,2]. A great number of health apps are already being used by millions of people, and several reports have pointed out the potential of these apps and digital technologies to deliver the right information to the right people at the right time. Health apps can enhance the safety, effectiveness, and efficiency of care by promoting health, contributing to the prevention and treatment of diseases, facilitating patients’ equal access to personalized health care, and engaging patients in their own care. They can also respond to unmet patient needs, enable better coordination of care, exploit the possibilities of remote health promotion and remote care, and strengthen the resilience and sustainability of health systems [3-5]. However, not all health apps provide sufficient evidence to support their claims, putting users at risk and calling into question their quality [6]. This creates a need for robust evaluation and regulatory oversight [3,4].

The European Union (EU) Medical Device Regulation (MDR) mandates a conformity assessment and related Conformité Européenne (CE) mark for all medical devices, including software, and is therefore applicable to medical apps [7-9]. However, while the MDR ensures patient safety, it produces merely a CE mark, not a quality report. Additionally, Notified Bodies are only involved in the evaluation of class IIa, IIb, and III medical devices; class I medical devices are not subject to third-party assessment. Wellness apps, on the other hand, fall outside the scope of the MDR, and digital marketplaces do not require robust evaluations [6]. As a result, health apps available on the market largely lack essential quality information apart from the user star ratings in app stores, which are known to be a poor indicator of quality [10]. To address this situation, several countries and regions in Europe and worldwide have developed assessment frameworks for health apps [11]. These frameworks assess characteristics beyond the MDR, such as data privacy, and provide more assessment information than a CE mark to support decision-making. The TIC Salut Social Foundation (FTSS, Catalan abbreviation), within the Ministry of Health of Catalonia (Spain), was in 2017 one of the first European health authorities to develop such an assessment framework [12,13]. With the emergence of more frameworks, a lack of cross-country harmonization has been introduced, leading to a significant duplication of app assessment efforts for manufacturers and health care systems, causing confusion among app users [2]. In addition, despite the widespread interest in the use of health apps across Europe, even authorities with relatively well-established frameworks encounter challenges with efficient implementation [11].

The World Health Organization (WHO) has called on health authorities and researchers to establish a common methodology for evaluating health apps, highlighting the need for these evaluations to become a standard practice rather than an exception [3]. Founded upon the premise that international harmonization could highly benefit the integration of apps in health care systems [2], the European Commission commissioned the European Committee for Standardization (CEN) to develop a Technical Specification (TS) for quality and reliability of health and wellness apps, which was achieved in collaboration with the International Organization for Standardization (ISO). CEN ISO/TS 82304‐2 “Health software—Part 2: Health and wellness apps—Quality and Reliability” (henceforth referred to as 82304‐2 or the TS) was published in 2021 [1,2,14]. As a next step, the EU-funded Label2Enable project (2022‐2024) has been dedicated to facilitating the adoption of this TS as the common assessment framework within the EU and potentially beyond. The project has iteratively cocreated the Label2Enable handbook for app assessment with 82304‐2 and the Label2Enable certification scheme [15].

### Objective

The objective of this study was to generate a systematic comparative analysis of the FTSS assessment framework for health apps with the 82304‐2 framework, to identify all new and relevant concepts distinct to 82304‐2 and all Catalonia-specific concepts addressed by the FTSS framework, and to adequately consider and allow proper integration of 82304‐2 in Catalonia. Our broader aim was to provide this methodology to health authorities in other regions and countries to make informed decisions about the adoption and integration of 82304‐2 or other evaluation frameworks.

## Methods

### Study Design

**Figure 1.**
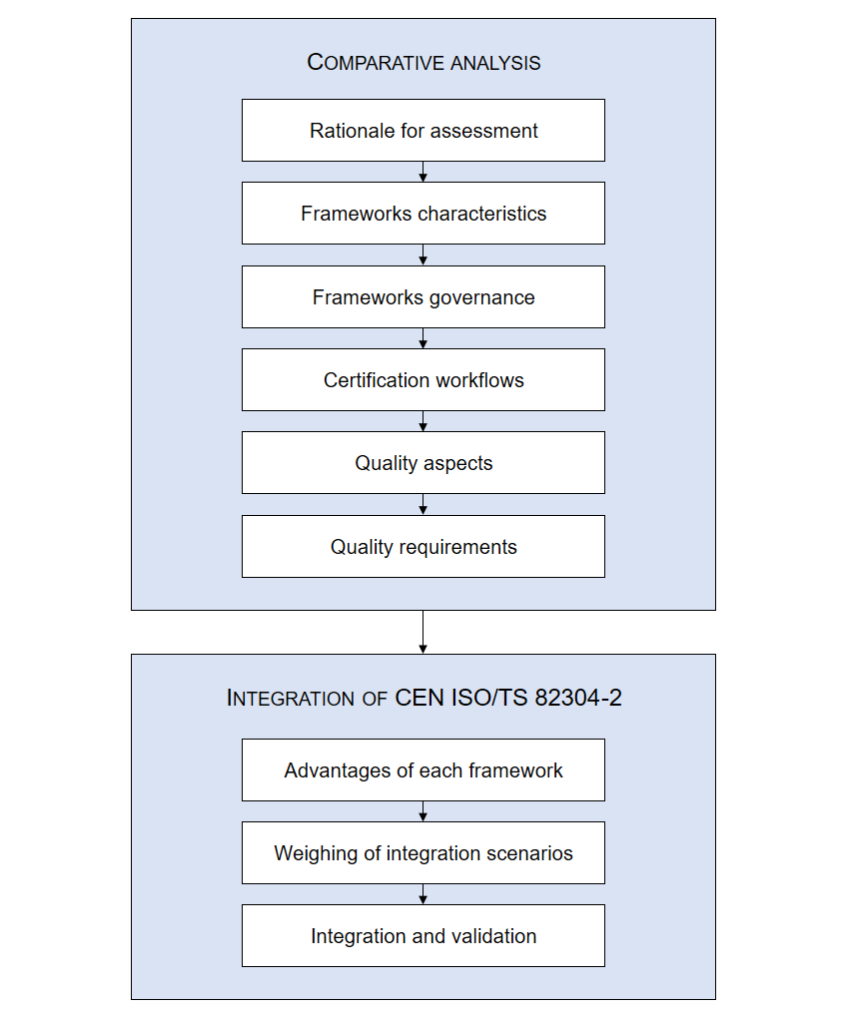
Overview of the methodology used in this study.

The project was carried out in 2 parts, as depicted in Figure 1. The first part, a comprehensive comparative analysis of both assessment frameworks, is divided into 6 sections. This part aimed to identify the characteristic traits and differences of each assessment framework. The second part is divided into 3 sections and involves considering the integration of 82304‐2 in Catalonia, weighing its advantages and disadvantages, and carrying out the decision made.

### Comparative Analysis

The comparative analysis was conducted using a mixed methods approach, comprising a qualitative case study, given its capacity to capture complexity, and a quantitative analysis. This methodology [16] was adapted from a comparative analysis of assessment frameworks for sustainability [17]. The analysis addressed qualitatively and descriptively the (1) rationale for assessment, (2) assessment framework characteristics, (3) assessment framework governance, (4) certification workflows and techniques used, (5) quality aspects and subaspects of each framework, and (6) quality requirements (or criteria).

To enable a quantitative analysis of the FTSS and 82304‐2 requirements shared across the 2 assessment frameworks (6), first, all concepts and subconcepts assessed in each of the 82304‐2 and FTSS requirements were identified by 2 researchers (BLC and CPB). The concepts and subconcepts were organized within the 82304‐2 subaspects (“Health requirements,” “Health risks,” “Ethics,” “Health benefit,” “Societal benefit,” “Accessibility,” “Usability,” “Privacy,” “Security,” “Technical robustness,” and “Interoperability”). In cases where multiple requirements addressed the same overarching concept, distinctions were made to recognize when they assessed different subconcepts. For the case of FTSS requirements, concepts were extracted both from the FTSS quality requirements and from the FTSS assessment submission form. In some instances, individual requirements were broken down into 2 distinct subconcepts to account for a proper comparison. Similarly, multiple requirements were grouped under the same concept and subconcept when applicable.

To compare the concepts and subconcepts of both frameworks, first, the obtained 82304‐2 subconcepts were searched within the FTSS requirements. The process was repeated in the opposite direction for FTSS subconcepts in 82304‐2 requirements. When finding matches, the original requirements of 82304‐2 and FTSS were compared. In total, 3 outcomes were possible: 0=“no match,” 0.5=“partial match,” and 1=“full match,” considering aspects such as the intended assessment goal, the level of detail required, and any differences in scope. Further detail as to the degree and type of partial match was deemed unnecessary for this paper, as the distinction between “no match” and “partial match” was primarily used to determine which FTSS requirements to discuss with the lead expert of 82304‐2 (PH) to ensure content was not accidentally overlooked and subconcepts were adequately identified as “partial match” or “no match.” Consensus on this identification was reached for all concepts and subconcepts.

Then, overall match scores were computed. The sum of all full matching subconcepts and partial matching subconcepts*0.5 was labeled “Both” (meaning that both frameworks assess this subconcept), the sum of all not matching subconcepts and partial matching subconcepts*0.5 applicable to 82304‐2 as “82304‐2 only,” and the sum of all not matching subconcepts and partial matching subconcepts*0.5 applicable to FTSS as “FTSS only.” Overall match scores were calculated as the sum of all full matching subconcepts and partial matching subconcepts*0.5 divided by the total number of subconcepts. The values were then converted to a percentage and plotted with R.

### Integration of CEN ISO/TS 82304-2

The second part of our study consisted of an analysis of the results of the comparative analysis to decide on the integration of 82304‐2 into the FTSS assessment framework. The analysis was carried out by 2 researchers (BLC and CPB). Integration would imply using the concepts and subconcepts identified in 82304‐2 for the assessment of health apps’ quality in Catalonia.

First, the advantages and disadvantages of each assessment framework and their certification workflow were identified and discussed. Next, the integration scenarios were considered—whether (1) to adopt the Label2Enable 82304‐2 certification scheme and related handbook adding only Catalonia-specific requirements, (2) to adopt 82304‐2 requirements into the FTSS framework (using the phrasing of 82304‐2), (3) to adapt 82304‐2 requirements according to FTSS needs, or (4) to maintain the current FTSS assessment framework in Catalonia.

### Ethical Considerations

Approval from an ethics committee was not required under Catalan and Spanish regulations, as the study did not involve human participants, personal health data, or clinical interventions. According to Law 14/2007 on Biomedical Research (Ley 14/2007 de Investigación Biomédica) and the guidelines of the Research Ethics Committee of Catalonia (Comitè d’Ètica de la Investigació amb Medicaments de Catalunya, CEIm), studies based solely on document analysis and framework comparison do not require ethical approval. Additionally, no identifiable personal or patient data were processed.

## Results

### Comparative Analysis

#### Overview

Key findings from the comparative analysis included a significant alignment between the FTSS assessment framework and 82304‐2, with quite similar rationales for assessment, framework governance, and certification workflows. The main difference between the frameworks was the app usage–based assessment (FTSS) versus evidence- and app usage–based assessment (82304‐2). Also, the 82304‐2 framework provided many concepts new to FTSS, which were all considered relevant.

#### Rationale for Assessment

In Catalonia, at the beginning of 2017, the Government requested the establishment of the FTSS assessment framework to ensure the quality of health apps used within the Catalan health care system and to promote the use of these health apps [18]. The development of 82304‐2 was commissioned by the European Commission to tackle the scattered assessment landscape with its many different criteria that struggle to scale, duplicate work, produce contradicting results, create challenges for manufacturers, and generally ignore international standards applicable to health apps [1,2].

Both the FTSS and 82304‐2 rationale for assessment included the large scale (hundreds of thousands of health and wellness apps, millions of downloads), concerns about the risks (many apps collect sensitive personal information and provide health advice that may not be supported by any evidence; app stores have limited evaluations; only some apps fall under the MDR), and opportunities missed (apps that are proven effective in addressing, for example, unhealthy lifestyles, chronic diseases, access to and affordability of health and care are not necessarily adopted at scale and reimbursed).

#### Framework Characteristics

Table 1 summarizes and compares the main characteristics of each assessment framework [1,2,12,15,18-22]. More detailed information can be found in Multimedia Appendix 1.

**Table 1.** Comparison of the main characteristics of the TIC Salut Social Foundation (FTSS) assessment framework and CEN ISO 82304‐2 Technical Specification (TS or 82304‐2).

	FTSS assessment framework	CEN ISO/TS 82304‐2
Year of publication	2017	2021
Scope	Catalan health care system	International (initiative Europe)
Scientific evidence	The FTSS framework was based on clinical guidelines and research papers (with input of relevant stakeholders in Catalonia).	The 82304‐2 framework was based on a Delphi study with 83 experts from 8 stakeholder groups and 6 continents. The Label2Enable 82304‐2 handbook “pass/fails,” and subquestions are informed by international scientific studies on health app quality and risks. The Label2Enable project included many studies to assess and fine=tune compatibility of 82304‐2 related products with mostly European multistakeholder needs.
Consensus	Relevant stakeholders in Catalonia	Key stakeholders, mostly European
Product information	Obtained from the FTSS assessment submission form	1 aspect: product information
Quality aspects	4 quality aspects: Clinical contents and functionality Usability, accessibility, and design Security and privacy of data Technological robustness	4 quality aspects: Healthy and safe Easy to use Secure data Robust build
Quality requirements	120 quality requirements	74 quality requirements within the 4 quality aspects (81 requirements with the additional 7 requirements in “Product information”)
Score-impacting requirements	Maximum 120	Maximum 67
Structure of each requirement	Affirmative sentence with: Description Levels of obligatoriness	Question with: Condition Purpose Response options Evidence Notes
Assessed apps	20 for testing, 55 for assessment, 23 as smoke test	35 for testing (11 in development of 82304‐2, 24 in development of the Label2Enable 82304‐2 handbook)
Certified apps	14 out of 55 assessed	0 (given testing phase)
Results communication	Certification seal + internal report	Quality label + quality report

#### Framework Governance

**Figure 2.**
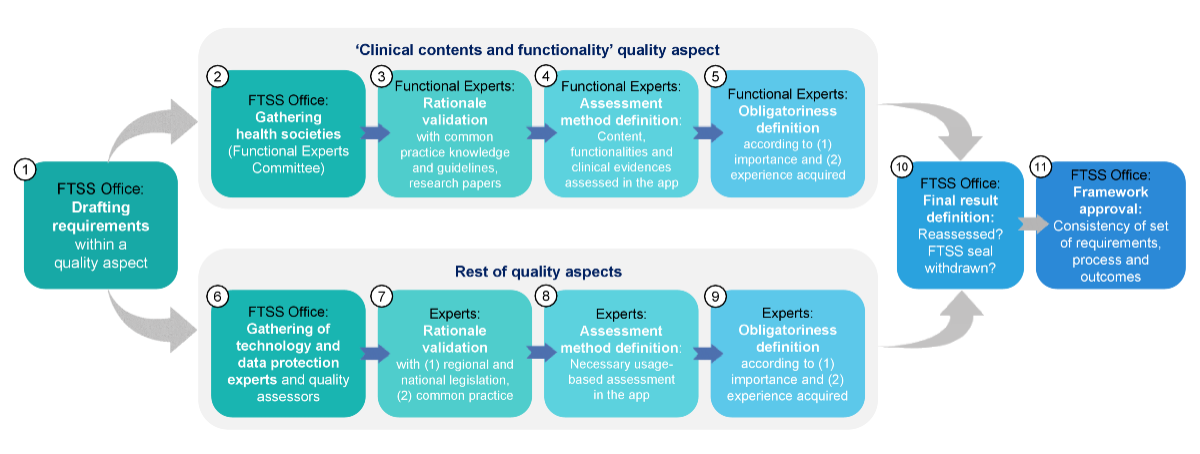
Methodology of definition and maintenance of each quality aspect within the TIC Salut Social Foundation (FTSS) assessment framework.

**Figure 3.**
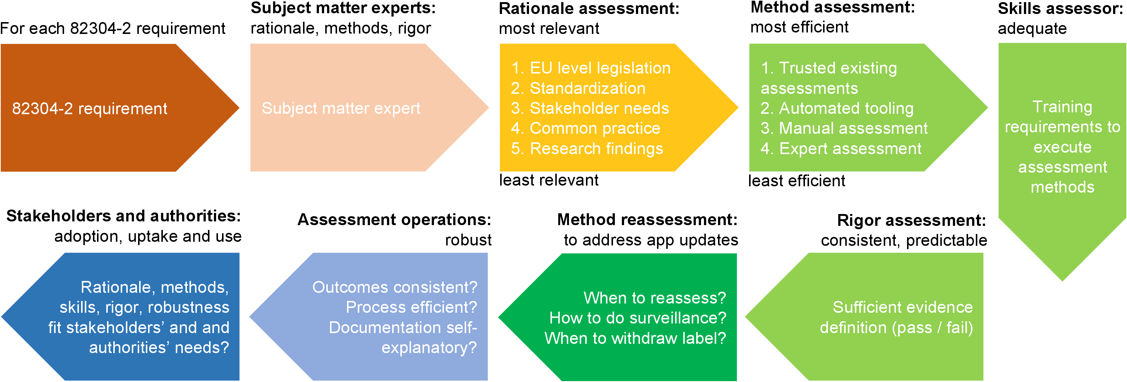
Methodology to cocreate the Label2Enable handbook for CEN ISO/TS 82304‐2 (82304‐2) for use by certified app assessment organizations. EU: European Union.

Both FTSS and Label2Enable have a stakeholders and experts organization to help approve, maintain, and improve their framework. Both FTSS and Label2Enable have defined a methodology of maintenance, with many steps overlapping (see Figure 2 for FTSS and Figure 3 for Label2Enable). Table 2 outlines and compares these maintenance methodologies. A broader explanation of each governance process can be found in Multimedia Appendix 2.

**Table 2.** Comparison of the main traits of the maintenance process of the TIC Salut Social Foundation (FTSS) assessment framework and the CEN ISO/TS 82304‐2 (82304‐2) Label2Enable handbook, outlined in Figures2  3 (and referenced in this table).

	FTSS assessment framework	CEN ISO/TS 82304‐2: Label2Enable handbook
Maintenance cycle	Maintained every 2-3 years	Intent to maintain annually and more frequently if needed
Methodology (steps 1 and 2 of Figures2 3)	Experts to consult recruited per quality aspect	Experts to consult recruited for each quality requirement
Experts (step 2 of Figures2 3)	Societies of health professionals [23], technology and data protection experts, quality assessors	Subject matter experts and key stakeholders
Rationale validation (step 3 of Figures2 3)	Legislation, stakeholder needs, and common practice	EU level legislation, stakeholder needs, common practice, standardization, and scientific research findings
Assessment method (step 4 of Figures2 3)	Decided for each requirement. Can include expert and trusted existing assessments	Evolves the assessment method and efficiency from expert to manual, where applicable, using automated and trusted existing assessments
Obligatoriness definition (step 5 of Figure 2)	Establishes which requirements are mandatory to obtain the certificate, which can be reviewed over time	Establishes which requirements are mandatory to qualify for the quality label, which can be reviewed over time
Assessors training (step 5 of Figure 3)	Not contemplated by FTSS as assessors have previous knowledge and experience	Considers the needed skills of the assessor and its training based on the assessment methods
Evidence definition (step 6 of Figure 3)	No	Defines the specific evidence needed to pass or fail for each requirement
Impact (step 9 of Figure 3)	Not evaluated by FTSS per se, but the FTSS framework was built by considering its impact	Explores the “compatibility” with what key stakeholders and authorities consider useful (and proportionate)

#### Certification Workflows

**Figure 4.**
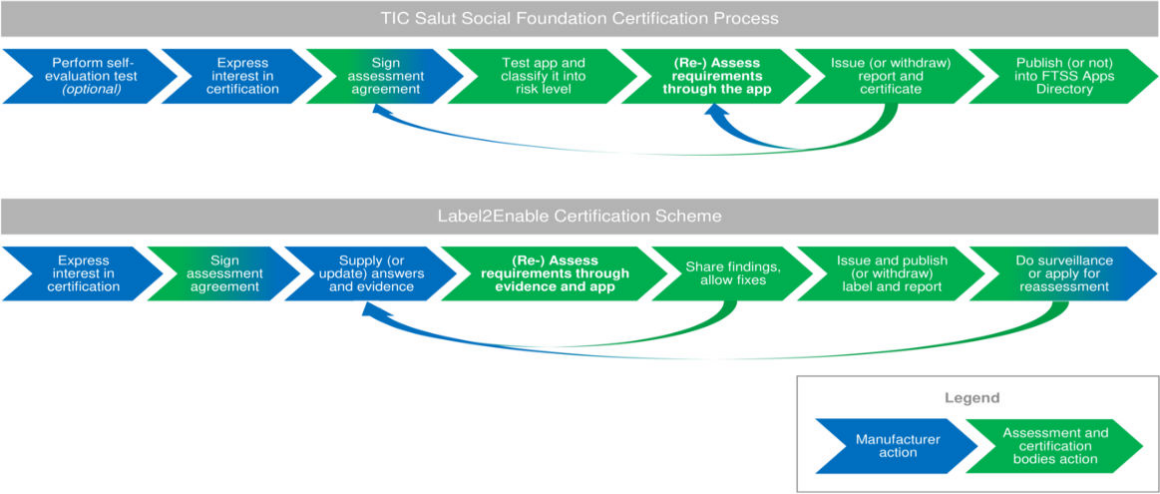
Overview of the certification workflows of TIC Salut Social Foundation (FTSS) (above) and the Label2Enable certification scheme (below).

The certification workflows of FTSS [21] and 82304‐2 (as defined by Label2Enable) are quite comparable (Figure 4). The main difference between the two is that the FTSS assessment is mostly app usage–based, while the Label2Enable 82304‐2 assessment is evidence- and app usage–based. In FTSS, assessors use the app and analyze its content and functionalities to decide on the pass or fail of each requirement. Following the Label2Enable 82304‐2 handbook, the manufacturer must supply evidence for each quality requirement that they claim to meet. Subsequently, a conformity assessment body assesses the evidence to determine the pass or fail, also checking the app. Other differences are that FTSS includes an optional self-evaluation test [24] and publishes certified apps in the FTSS directory. Label2Enable intends to provide a verification service of the label it supplies and proposes to generate a directory. For more detail about the certification workflows, see Multimedia Appendix 3.

#### Quality Aspects

**Figure 5.**
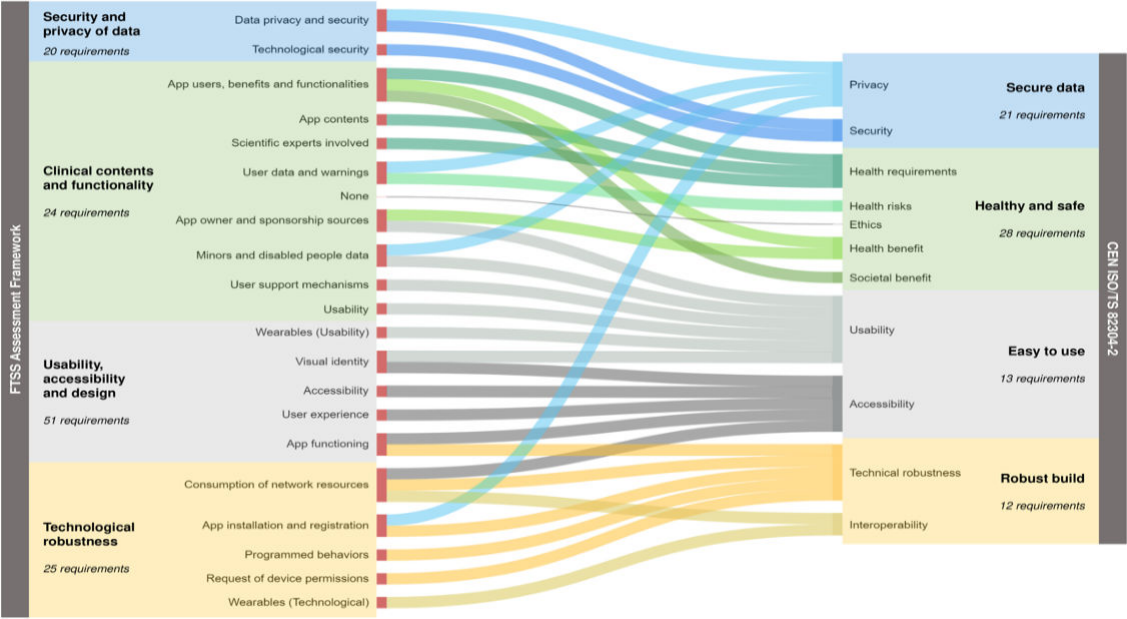
Matches found between the quality aspects and subaspects of the TIC Salut Social Foundation (FTSS) and the CEN ISO/TS 82304‐2 (82304‐2) assessment frameworks. The 4 quality aspects of FTSS (eg, “Security and privacy of data”) are displayed in bold on the left, and the 4 quality aspects of 82304‐2 (eg, “Secure data”) in bold on the right. The colored boxes also contain the number of quality requirements within the quality aspect and the subaspects in which these requirements are clustered. The similar background colors (blue, green, grey, and yellow) indicate a match of the quality aspects between the 2 frameworks. The lines in the middle connect the concepts within the FTSS and 82304‐2 quality requirements that were similar. The color coding of the connecting lines differs per 82304‐2 subaspect it originates from and is merely used to support easy recognition of the distribution of the 82304‐2 concepts across FTSS subaspects. Note that this is a qualitative analysis; thus, the thickness of the connection lines is not numerically representative.

The FTSS and the 82304‐2 assessment frameworks both contain 4 overarching quality aspects [2,20]. Based on the semantics of each aspect and the concepts and subconcepts assessed in each, a match between the aspects of the 2 frameworks was observed (Figure 5). On a more detailed level, differences in distribution applied, which highlighted a need to consider assessment frameworks in its entirety in a comparative analysis, instead of merely considering semantically similar subsections. The organization of 82304‐2 seems a bit more logical; FTSS has some scattered requirements when compared to 82304‐2. For example, the quality aspect “Clinical contents and functionality” of FTSS is broader than “Healthy and safe” of 82304‐2, with many of FTSS’ subaspects connecting to 82304‐2’s “Easy to use” and some to “Secure data.” “Technological robustness” is also broader than “Robust build.” The concepts gathered in 82304‐2 subaspect “Privacy” are distributed among 4 FTSS subaspects, from 3 different quality aspects. All in all, concepts assessed are fairly common in both frameworks, but wording used for aspects and subaspects within FTSS and 82304‐2 tends to differ, with some exceptions, such as “Usability” and “Accessibility.”

#### Quality Requirements

**Figure 6.**
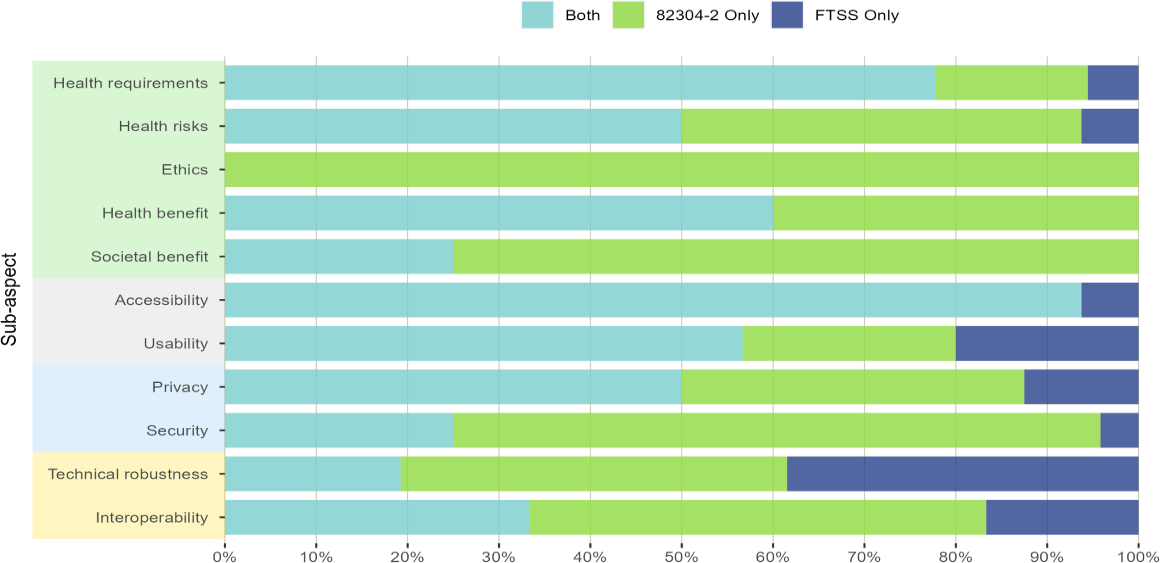
Percentage match of subconcepts between the CEN ISO/TS 82304‐2 (82304‐2) and TIC Salut Social Foundation (FTSS) assessment framework clustered according to the 82304‐2 subaspects. Colored boxes framing the subaspects indicate the 82304-2 aspect to which they originally belong to: “Healthy and safe” (green), “Easy to use” (gray), “Secure data” (blue), and “Robust build” (yellow).

To enable a comparison of all the 82304‐2 and FTSS quality requirements, in total, 78 different concepts and 97 subconcepts were extracted from all 74 quality requirements of the 82304‐2 framework and all 120 quality requirements of the FTSS framework combined. Overall, 48% (47/97) of the subconcepts of both frameworks were a partial or full match, 39% (37.5/97) were 82304‐2-specific (“82304‐2 only”) and 13% (12.5/97) FTSS-specific (“FTSS only”). The subaspect “Accessibility” had the highest match, with 94% (7.5/8) of subconcepts assessed in both frameworks, and FTSS addressing another 6% (0.5/8) (see Figure 6). Other subaspects such as “Health requirements,” “Health risks,” “Health benefit,” and “Usability” had at least 50% of subconcepts covered by both frameworks. The 82304‐2 requirements were found to cover most of the FTSS requirements, ranging from subaspects such as “Health benefit” and “Societal benefit” missing no concepts (0%) to “Technical robustness” missing a maximum of 38% (5/13). The most relevant “82304‐2 only” subaspect was “Ethics,” which is not assessed by FTSS. For other subaspects (“Societal benefit,” “Security,” and “Interoperability”), 82304‐2 covered at least 50% of the subconcepts.

For more detail, Table 3 provides the match scores obtained for each concept assessed in the frameworks. Note that this comparison has been performed with the original 82304‐2 rather than with the more detailed Label2Enable 82304‐2 handbook for certified app assessment organizations, which provides standardized subquestions to assess the 82304‐2 quality requirements and thus may add more subconcepts.

**Table 3.** Evaluation of the degree to which each concept and subconcept identified in the CEN ISO/TS 82304‐2 (82304‐2) and TIC Salut Social Foundation (FTSS) assessment framework was matched in the other framework. In total, 3 outcomes were possible: 0=no match, 0.5=partial match, and 1=full match. From the 2 frameworks combined, in total 78 concepts and 97 subconcepts were extracted.^a^

Concepts extracted within each subaspect	Subconcepts extracted (n)	Concepts match (sum of subconcepts match scores)		
		Both	82304‐2 only	FTSS only
Health requirements				
Intended users^b^	1	1	0	0
Age restrictions	1	1	0	0
Health issues^b^	1	0	1	0
Intended use^b^^,c^	1	1	0	0
Medical device^c^	1	1	0	0
Health professionals’ collaboration	2	1.5	0	0.5^d^
Literature used	2	1.5	0.5	0
Health risks				
Risks analysis	1	0	1	0
Risks control	2	0	2	0
Professional approval before use^b^	1	0.5	0.5	0
Risks communication	1	1	0	0
Contraindications and limitations	2	1.5	0	0.5^d^
Safety concerns and incidents	1	1	0	0
Ethics				
Ethical challenges	1	0	1	0
Approval from ethics board	1	0	1	0
Health benefit				
Health benefit^b^	1	1	0	0
Health interventions	1	0	1	0
Financial cost for users	1	0	1	0
Need of health professional support	1	0.5	0.5	0
Evidence for health benefit	2	1.5	0.5	0
Maintenance of health information	1	0.5	0.5	0
Sources for health information	1	1	0	0
Funding	1	1	0	0
Advertising	1	0.5	0.5	0
Societal benefit				
Societal benefit	1	0.5	0.5	0
Evidence for societal benefit	1	0	1	0
Accessibility				
Perceptibility	2	2	0	0
Operativity	2	2	0	0
Understandability	2	2	0	0
Robustness	1	0.5	0	0.5^d^
Age appropriateness	1	1	0	0
Usability				
Design taking users into account	2	1.5	0	0.5^d^
Users involved in design	1	0	1	0
User-centered evaluation	1	0	1	0
Measures to avoid misuse	3	2	0	1^e^
App information communication	3	2	0.5	0.5^d^
Instructions for use	2	1.5	0	0.5^d^
User support when experiencing problems	1	1	0	0
Mechanisms for network and systems problems avoidance	1	0.5	0	0.5^d^
Usability research for improvements	1	0	1	0
Privacy				
Processing of personal data^b^^,c^	1	0.5	0.5	0
Processing of personal health data^b^	1	0	1	0
Data minimization	1	1	0	0
Data retention policy	2	1.5	0	0.5^f^
Privacy statement for users	1	1	0	0
Data processing of minors and severely disabled people	1	0.5	0	0.5^d^
Secure contracts with data processors	1	0	1	0
User approval for data use and opt-in default	2	1.5	0	0.5^d^
Data protection officer	1	0	1	0
Security incident response procedures	1	0	1	0
Security				
Security management implementation	1	0	1	0
Information security risk assessment	1	0	1	0
Secure by design process	1	0	1	0
Security of third-party software	1	0	1	0
Security of source code	1	0.5	0.5	0
Organizational measures for legitimate processing of data	1	0	1	0
Security with user authentication	2	1.5	0	0.5^d^
Encryption of data stored	1	1	0	0
Management of security vulnerabilities	1	0	1	0
Regular testing of app security	1	0	1	0
Security policy for users	1	0	1	0
Technical robustness				
Product requirements	1	0	1	0
Software development with standards and methods	1	0	1	0
Secure coding standard	1	0.5	0.5	0
Configuration management plan	1	0	1	0
Dealing with increase in demand	1	1	0	0
Validation and verification plan	1	0	1	0
Release and deployment process	1	0.5	0.5	0
Maintenance process	1	0.5	0.5	0
General functioning	2	0	0	2^d^
Functioning with device events	1	0	0	1^e^
Use of device resources	1	0	0	1^e^
Use of network resources	1	0	0	1^e^
Interoperability				
Functioning with wearables	2	1	0	1^g^
Guides and specifications for all APIs^h^	1	0.5	0.5	0
Guides and specifications for terminologies	1	0	1	0
Validation of data transferred via APIs	1	0.5	0.5	0
Export of app data	1	0	1	0

a Concepts and subconcepts of each requirement in each assessment framework have been extracted and organized according to 82304‐2 subaspects but include both 82304‐2 and FTSS requirements.

b This subconcept is not score-impacting in 82304‐2.

c This subconcept is not score-impacting in the FTSS framework.

d This “FTSS only” subconcept is now included in the subquestions of the Label2Enable 82304‐2 handbook.

e This “FTSS only” subconcept is now partially included in the subquestions of the Label2Enable 82304‐2 handbook. This means that the subconcept is included, but with a lower level of detail. For example, the Label2Enable 82304‐2 handbook checks whether measures to avoid misuse are in place, but not all measures that FTSS assesses have been included. In most cases, the Label2Enable consortium considered this level of detail not relevant.

f This “FTSS only” subconcept is context-specific for Catalonia or Spain. It is not for 82304‐2 uptake.

g This “FTSS only” subconcept is scope-expanding, that is, it goes beyond the definition of a health app assessment framework, at least for 82304‐2. Currently, it is not for 82304‐2 uptake.

h API: application programming interface.

Both for the “82304‐2 only” and the “FTSS only” subconcepts, it is important to consider that these subconcepts may not be relevant or may be of lesser importance in the other assessment framework. Of the initial 13% (12.5/97) “FTSS only” subconcepts, 7% (7/97) were in the meantime included in the subquestions of the Label2Enable 82304‐2 handbook as considered relevant, 1% (1/97) was scope-expanding and thus was not for 82304‐2 uptake, and 1% (0.5/97) was related to specific Catalan and Spanish regulations, also not for 82304‐2 uptake. The remaining 4% (4/97) “FTSS only” subconcepts were specific to app usage–based assessment of the technical robustness of the app, labeled as rigor-enhancing subconcepts, and not included in the Label2Enable 82304‐2 handbook, as the evidence-based assessment was considered sufficient.

### Integration of CEN ISO/TS 82304-2

#### Advantages of Each Framework

In general, both assessment frameworks were found to be quite similar. The international applicability, foundation in standardization, scientific evidence, concepts covered, and maintenance of 82304‐2 and Label2Enable make the framework distinctive in rigor and capable of enabling harmonization and preventing a duplication of efforts. In addition, the 82304‐2 health app quality label and report, the more detailed version of the 82304‐2 health app quality label that was cocreated with health care professionals to support decision-making on a health app, are expected to be useful for potential users, health professionals, and health systems to increase the willingness to use health apps. It is remarkable of FTSS; nevertheless, that the assessment of their framework is supported by societies of health professionals collaborating in the Functional Experts Committee, and that they have more years of experience in their assessment. On the other hand, Label2Enable tested with medical societies the usefulness of the health app quality report in providing guidance on health apps. An article with the European Society of Cardiology established that 82304‐2 covered all specified requirements for apps in the 4 intended uses and 3 health issues investigated [25].

Regarding the assessment itself, combining evidence- and app usage–based assessment, as defined in the Label2Enable 82304‐2 handbook, was considered by the researchers to provide the most advantages in rigor. The objectivity and consistency of the assessment are higher in Label2Enable due to the specific terminologies (eg, ‘intended use,’ ‘intended user’), and the clarity for each quality requirement of what is needed to pass, although it is valuable of FTSS that their framework calculates the risk level of the app to determine the number of mandatory requirements. CEN ISO/TS 82304‐2 has a similar approach, with at most 67 score-impacting requirements, depending on, among other intended uses, whether an app includes health information, processes personal data, and is interoperable. On a global level, 4 requirements are mandatory to qualify for an 82304‐2 label; on an EU level; more requirements are mandatory based on an alignment with EU-level legislation and values. Additionally, FTSS has a self-evaluation (readiness) test for manufacturers, which was recommended to be implemented similarly for 82304‐2.

Considering the quality requirements themselves, the most important dimension to decide on integration, 82304‐2 covered 87% (84.5/97) of the subconcepts of both frameworks (47/97 (48%) “Both” plus 37.5/97 (39%) “82304‐2 only”). By having added 7 “FTSS only” subconcepts to the subquestions, the current Label2Enable 82304‐2 handbook contains 100% of the EU-relevant requirements of 82304‐2 and FTSS assessment frameworks. The remaining 5.5 FTSS subconcepts (1 scope-expanding, 0.5 context-specific, and 4 rigor-enhancing) are specific to the FTSS assessment framework and can be assessed separately on top in Catalonia. Overall, 82304‐2 and the Label2Enable certification scheme provide many advantages; in short, a broader, more objective, pan-European, evidence-based assessment framework that was considered to provide an enhanced value.

#### Weighing of Integration Scenarios

Given the advantages that 82304‐2 proved to provide, scenario (4) was disregarded, making the remaining viable integration scenarios (1) to adopt the Label2Enable 82304‐2 certification scheme and related handbook, adding Catalonia-specific additional requirements on top, (2) to adopt the 82304‐2 requirements into the FTSS framework (using the phrasing of 82304‐2), or (3) to adapt 82304‐2 requirements according to our needs.

The Label2Enable consortium proposes an EU-level assessment via the Label2Enable certification scheme and 82304‐2 handbook, with each country (or region) then assessing only the requirements specific to the country (or region), being context-specific, scope-expanding, and rigor-enhancing requirements. For Catalonia and manufacturers involved, cross-country recognition would simplify and accelerate the certification process. Thus, FTSS sees value in directly using the Label2Enable certification scheme (scenario 1) once it is operational and 82304‐2 health app quality labels and reports are issued.

However, as the Label2Enable certification scheme is not yet operational, and the Government of Catalonia needs to carry on with app assessments and is interested in already improving its framework, we decided to start by implementing 82304‐2 ourselves and adapting some requirements according to our needs (mix of scenarios 2 and 3). This allowed the Label2Enable certification scheme to follow its process of implementation in Europe while updating the FTSS framework.

#### Integration and Validation

All 81 82304‐2 requirements were incorporated in the new FTSS framework, using different integration mechanisms based on the match scores. For unmatched 82304‐2 concepts and subconcepts, the 82304‐2 requirements were adopted as is. For full matches, FTSS-related subtleties were added to the 82304‐2 requirements. Of the partially matched FTSS subconcepts, 8 could be added to existing 82304‐2 requirements. Next, the remaining 3 partially matched FTSS subconcepts, the 7 unmatched “FTSS only” subconcepts, and 1 new concept originating from a consultation of the newest regional guidelines [26] and Catalan subject matter experts, as per the methodology outlined in Figure 2, were phrased as a question to create 11 new quality requirements for the new FTSS framework. The entire set of requirements was translated to Catalan using plain language.

Subsequently, 5 of these 11 new requirements were included in the Label2Enable 82304‐2 handbook. The 6 remaining FTSS-specific requirements included 1 scope-expanding requirement (wearables), 4 rigor-enhancing requirements (additional app usage–based assessment), and 1 context-specific requirement (ensuring that apps developed within the Ministry of Health of Catalonia contain the required visual identity).

FTSS has already developed a new web-based form for submitting the necessary information and evidence for assessment. Assessment results will be tested in the following months, and the intent is to present the results with a quality label very similar to that of 82304‐2. Once the Label2Enable 82304‐2 handbook is operational and 82304‐2 quality labels and reports are issued, scenario 1 will become feasible. Then, the new Catalan assessment framework would consist of the 82304‐2 label and report with 81 requirements and its added Label2Enable 82304‐2 handbook subquestions, plus 6 additional requirements assessed by FTSS. That would mean the 82304‐2 label covers 93% (81/87) of the Catalan assessment, with FTSS having to assess only 7% (6/87).

## Discussion

### Principal Findings

We successfully achieved our objective of generating a systematic and comprehensive comparative analysis of the FTSS assessment framework for health apps with the 82304‐2 framework. We identified 78 concepts and 97 subconcepts; 37.5 of these were new or partially new subconcepts from 82304‐2, all relevant to Catalonia, and 12.5 were FTSS-specific. All 81 82304‐2 requirements were incorporated in the new FTSS framework. FTSS subtleties and partially matched subconcepts were added to the existing 82304‐2 requirements where suitable. The remaining FTSS-specific concepts and subconcepts were rephrased as questions to create 11 additional quality requirements. Of these 11 requirements, 5 were integrated in the Label2Enable 82304‐2 handbook. Once the Label2Enable 82304‐2 handbook is operational and can be adopted, the remaining 6 requirements will be assessed on top of the existing 81 82304‐2 requirements. Of these 6, 1 requirement is scope-expanding, 4 are rigor-enhancing, and 1 is context-specific.

The methodology used enabled us to adequately consider and achieve proper integration of 82304‐2 in Catalonia. We believe that this comparative methodology can serve as a useful model for health authorities in other regions and countries considering or seeking to integrate 82304‐2 into their own assessment framework and adopt the Label2Enable 82304‐2 handbook. In such cases, we expect that the comparison of the quality requirements is key. In our case, extracting the concepts and subconcepts from the FTSS and 82304‐2 quality requirements highly increased the reliability of the percentage matches obtained. The percentages obtained for each subaspect, the relevance of all “82304‐2 only” concepts to Catalonia, as well as the low percentage of Catalonia-specific requirements to be assessed on top, were particularly useful for decision-making and necessary for actual 82304‐2 integration.

### The Results From a Theoretical Perspective

Rogers’ diffusion of innovation theory [27] was foundational in the Label2Enable project and is of value given our broader aim to provide this methodology for health authorities elsewhere to consider integrating 82304‐2 or other evaluation frameworks. Rogers’ theory acknowledges 5 attributes that influence rate of adoption of an innovation. In this case, 82304‐2 can be considered an innovation. The first and main attribute, according to Rogers, and in our experience, is relative advantage, the degree to which 82304‐2 is perceived as better than the current FTSS framework. Overall, 82304‐2 has been observed to provide a positive relative advantage in comparison with the current FTSS framework. First, 82304‐2 entails a more structured, better maintained, standards- and evidence-based framework, with additional value-adding concepts and subconcepts to assess the quality of a health app compared to the FTSS framework. Second, 82304‐2 is an international standardization effort, and the Label2Enable 82304‐2 handbook adopts a pan-European perspective, making it a more robust and scalable solution for app assessment across borders. This broader scope allows for the harmonization of assessment requirements, which is especially beneficial given the current lack of cross-country standardization and efficiency and scalability challenges in health app assessments. This advantage cannot be reached with a regional framework. Thirdly, the Label2Enable 82304‐2 handbook’s evidence-based assessment approach on top of app usage–based assessment with clearly defined terminologies and definitions of what is needed to pass was evaluated favorably by FTSS. For this reason, the Label2Enable 82304‐2 handbook will be adopted, and all 82304‐2 requirements were incorporated in the new FTSS framework. FTSS subconcepts not covered by 82304‐2 were maintained as additional requirements, as suggested by Frey et al [28] and already operationalized by the Australian eHealth Agency’s framework [29].

Rogers’ second attribute is compatibility, the degree to which integration of 82304‐2 is perceived as consistent with the existing known values, experiences, and needs of Catalonia. Beyond our decisions detailed in the previous paragraphs, we propose multistakeholder feedback to assess compatibility and specify expected benefits, challenges, and the practical implications of integration from a multistakeholder perspective, enhancing existing knowledge on the compatibility of 82304‐2 from a health authority perspective [30]. In June 2023, FTSS invited the members of the Functional Experts Committee, formed by different societies of health professionals, to discuss the value of 82304‐2. Careers of its 8 members can be found on the FTSS website [23]. They concluded that this certification could become greatly useful if it became mandatory for all health apps in the European market, and especially if it appeared in the apps marketplaces. This way, the quality of all published health apps would be ensured, and patients, professionals, and health care systems would use and recommend health apps with more confidence and reliability. Additionally, in a later meeting, they valued the Label2Enable health app quality report as a powerful tool to obtain relevant quality information about health apps and thus allow them to prescribe these apps. In the Label2Enable project, the manufacturer perspective was obtained through a discrete choice experiment with 41 manufacturers and a pilot study with 24 manufacturers. The first study showed that manufacturer willingness to pay for app assessment was primarily associated with the potential for integration of the health app into clinical guidelines and reimbursement or procurement [31]. In the second study, after having participated in a pilot 82304‐2 assessment, 87.5% (21/24) of the manufacturers responded positively to willingness to “do it again” [32]. The Catalan health care system currently considers making this new assessment free of charge for apps of interest.

Rogers’ third attribute is complexity, the degree to which 82304‐2 integration is perceived as relatively difficult to understand and use. Once the Label2Enable 82304‐2 handbook is operational and 82304‐2 quality labels and reports are issued, the new Catalan assessment framework would consist of the 82304‐2 quality label and report plus only 6 additional requirements assessed by FTSS (7%, 6/87). A further expansion of the 82304‐2 scope, as currently advocated for the upcoming 82304‐2 revision, would likely eliminate the need for assessing the scope-expanding requirement by FTSS, further reducing the Catalonia-specific workload to 6% (5/87). Uptake of 82304‐2 thus avoids considerable duplication of assessment efforts with similar requirements. Currently, however, as the Label2Enable 82304‐2 handbook was not yet available, we used an extensive analysis of 82304‐2 to consider and achieve proper integration. To operationalize the new framework, training of the assessors will be required. Although it is expected that this integration may carry more workload for assessors, at least in the beginning, the time investment was considered acceptable in view of the enhanced value of the new framework and quality of certified apps that combined promote health app use. To end with, it will be necessary to properly decide how the quality labels are used; for instance, which scores are considered sufficient for apps to be used within the Catalan health care system.

The final 2 of Rogers’ attributes, trialability, the extent to which 82304‐2 can be tested before its integration, and observability, the level to which 82304‐2 integration provides tangible results, were considered of lesser importance at this stage. Taking into account that Label2Enable has already performed tests with 24 apps with the handbook [32], trials were considered mostly covered. However, also considering the handbook evolved after testing with 24 apps, Catalonia will test the resulting assessment framework with a minimum of 6 apps (hospital and private apps) by mid-2025. This test will include an analysis of the time investment for both manufacturers and assessors and the elapsed time between the assessment request and the availability of the assessment results. Finally, regarding observability, we especially perceive great potential of 82304‐2 in increasing the trust in and willingness to use health apps among all stakeholders, supported by recent [25,30] and upcoming 82304‐2-related publications. Once certificates are issued, it will be possible to observe the actual increase in the degree of trust and—gradually—use. For now, with the new 82304‐2 integrated assessment framework in Catalonia in place, entities within the Ministry of Health of Catalonia have gained more trust in the assessment framework. They are offering guidelines on health app development [26,33] to all health care providers (primary care and specialized care) within the Catalan health care system, developed by FTSS in parallel with the new assessment framework. They are also using the new assessment framework to ensure the quality of all apps integrated within the electronic health record patient portal (La Meva Salut).

### Future Developments

This study was one of many studies within Label2Enable; some have already been published [25,30-32,34], and others are expected to be published later in 2025. Most of these studies aimed at assessing the needs of a specific stakeholder group, the compatibility of 82304‐2 with these needs, and how to further increase related outcomes. In total, 4 multistakeholder backcasting workshops were used to arrive at a multistakeholder road map toward the preferred future of digital health and care and the role of digital health quality labeling in it [35]. Part of this road map is a demonstrator phase in which the first 50‐100 health app quality labels and related, more detailed reports can be issued. In this effort, the inter-rater reliability, efficiency, clarity, and scalability of the Label2Enable 82304‐2 handbook for certified app assessment organizations and the multistakeholder usefulness of the 82304‐2 health app quality report, both cocreated in the Label2Enable project, can be tested at a larger scale and further fine-tuned. Multistakeholder effectiveness is expected to further support decision-making by authorities and other stakeholders on adopting 82304‐2.

### Practical Recommendations

Following findings from this study, the Label2Enable project, and the WHO [3,35,36], we made recommendations for other authorities and stakeholders who seek to capture the value of digital health products (Textbox 1).

Textbox 1. **Recommendations to other health authorities and stakeholders who seek to capture the value of digital health products.**

1. Build your digital health policy based on international policies and applicable regulations, and an understanding of how digital health products can support the aims and address the challenges of your health care system or context.
2. Assign and equip an entity for regulation, oversight, and implementation of health apps or the larger scope of digital health products. Avoid, if possible, scattering responsibilities across authorities.
3. Determine your common assessment framework, assessment organization, and assessment process instead of starting from scratch. Identify with local stakeholders and experts the potential need for additional context-specific, scope-expanding, and rigor-enhancing requirements. Consider using 82304‐2 and the Label2Enable 82304‐2 handbook, and, if you have a framework in place, consider following the methodologies used in this study to evaluate and integrate 82304‐2. If more semantic rigor is preferred for analyzing concept matches, semantic knowledge tools can be considered. Consider also comparing other traits of the frameworks if applicable.
4. Engage with stakeholders, ranging from related authorities, health care providers, health professionals, and patients to digital health manufacturers, to assess their needs in integrating health apps. For instance, manufacturers’ needs in meeting the assessment requirements; related authorities’ needs in meeting health care system goals; health care providers’, professionals’, and patients’ needs in selecting, integrating, and using apps and the related data; and enabling, capturing, and measuring the value of apps and their data for the individuals, health care system, and society at large.
5. Facilitate the use of quality health apps with transformation and support. For example, by integrating them into health care pathways and developing guidelines. Measure progress in integrating health apps, multistakeholder value added, and what is required to enhance that value.

### Conclusions

The methodology presented has allowed a systematic, comprehensive comparative analysis of Catalonia’s FTSS health app assessment framework and 82304‐2. The results informed the FTSS decision to integrate the 82304‐2 quality requirements to achieve a more comprehensive, evidence-based, standardized approach to health app assessment, and adopt the Label2Enable 82304‐2 handbook in the future. CEN ISO/TS 82304‐2 included many subconcepts new to FTSS, which were all considered relevant for Catalonia. The Label2Enable 82304‐2 handbook was found to be value-adding in rigor and consistency, and the remaining FTSS-specific requirements were few. These findings are expected to be indicative of the potential of 82304‐2 to contribute to solving the heterogeneous and complex landscape of assessment frameworks for health apps, making robust health app assessments common. FTSS highly encourages other authorities to undertake a similar evaluation or to wait until the Label2Enable 82304‐2 handbook is operational to adopt it in full.

## Supplementary material

10.2196/67858 Multimedia Appendix 1 Fundamental information and characteristics of the CEN ISO/TS 82304-2 and TIC Salut Social Foundation assessment frameworks.

10.2196/67858 Multimedia Appendix 2 Governance and maintenance of the CEN ISO/TS 82304-2 and TIC Salut Social Foundation assessment frameworks.

10.2196/67858 Multimedia Appendix 3 Certification workflows of the CEN ISO/TS 82304-2 and TIC Salut Social Foundation assessment frameworks.

## Abbreviations

82304-2: CEN ISO/TS 82304-2
CE: Conformité Européenne
CEN: European Committee for Standardization
EU: European Union
FTSS: TIC Salut Social Foundation (Catalan abbreviation)
ISO: International Organization for Standardization
MDR: Medical Device Regulation
TS: Technical Specification
WHO: World Health Organization
