# Supplementary material for: Integrating CEN ISO/TS 82304-2 in the Catalan Health App Assessment Framework: Comparative Case Study
Source: JMIR Mhealth Uhealth. 2025 Jun 4;13:e67858. doi: 10.2196/67858 (PMC12154936; doi:10.2196/67858)
Supplement: Multimedia Appendix 1 [file mhealth-v13-e67858-s001.docx]

**Multimedia Appendix 1. Fundamental Information and Characteristics of the CEN ISO/TS 82304-2 and TIC Salut Social Foundation Assessment Frameworks**

**TIC Salut Social Foundation Assessment Framework**

The TIC Salut Social Foundation (FTSS) assessment framework was established at the beginning of 2017 at the request of the Government of Catalonia to ensure the quality of health apps used within the Catalan healthcare system [1] and has been in use since [2], with 55 health apps assessed. These include CE-marked medical apps (mainly accessories of other medical devices, generally wearables such as glucometers), medical apps centred on a wide range of medical specialities and pathologies, and wellness apps.

The framework was based on clinical guidelines, research papers and developed through a broad consensus among relevant stakeholders, including technology experts, health professionals, that is a Functional Experts Committee with representatives of Societies of Medicine, Nursery, Psychology and Physical education, among others, health communication professionals, institutional authorities of Catalonia, third sector organizations, expert patients, and some representatives of the public. This multidisciplinary approach ensured that the framework assessed what health professionals and users valued in health apps, as well as what was necessary for the Catalan healthcare system [2,3]. To establish the framework, an initial test assessment was conducted on 20 apps without issuing any certification.

Currently, the FTSS framework contains 120 assessment requirements, or 114 requirements in the case of apps that do not connect to a medical device such as a wearable, organized in 4 quality aspects; see **Table S1**. Each requirement is defined with an affirmative sentence, accompanied by a description to guide both assessors and manufacturers, and its level of obligatoriness – mandatory, recommended or desirable –. The possible answers for each of the requirements are “Yes” (pass), “No” (fail) or “Not applicable”. The FTSS Certification Seal (**Figure S1**) is granted to all apps that pass all mandatory requirements in the framework [4,5].

**Table S1.** Aspects and sub-aspects of the TIC Salut Social Foundation (FTSS) assessment framework, along with the number of requirements in each aspect and sub-aspect (in parentheses) [4].

| **Aspect** | **Sub-aspects** |
| --- | --- |
| **Clinical contents and functionality**    ***24 requirements*** | - App users, benefits and functionalities (3) - App owner and sponsorship sources (3) - App contents (7) - Scientific experts involved (2) - User data and warnings (5) - Minors and disabled people data (2) - User support mechanisms (2) |
| **Usability, accessibility and design**  ***51 requirements*** | - Usability (9) - User experience (30) - Visual identity (5) - Accessibility (4) - Wearables (Usability) (3) |
| **Security and privacy of data**  ***20 requirements*** | - Data privacy and security (7) - Technological security (13) |
| **Technological robustness**  ***25 requirements*** | - App installation and registration (4) - App functioning (6) - Consumption of network resources (7) - Programmed behaviours (3) - Request of device permissions (2) - Wearables (Technology) (3) |


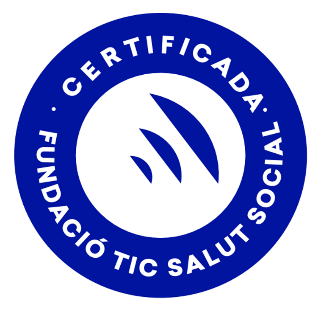


**Figure S1.** FTSS Certification Seal.

The level of obligatoriness of each of the requirements varies depending on the risk level of the app, as determined by assessors (more detail on the risk level can be found in Multimedia appendix 3); however, all requirements are score-impacting. For each aspect, a final score is obtained by summing and weighing individually the mandatory, recommended and desirable requirements (the higher the level of obligatoriness, the higher the weight) [5].

The requirements are publicly available (in Catalan) [4]. Certified apps are listed in the Digital Assets Directory (in Catalan, English and Spanish). Currently, this portfolio includes 14 certified apps [6], although 1 of these apps is no longer on the market. In addition, a final report with the score obtained is privately sent to the manufacturer. Interestingly, apart from the 55 assessed health apps, 23 health apps requested a smoke test (software build verification testing) while in development, only to obtain initial feedback. The FTSS Certification Seal could not be granted to these apps given their development stage.

**CEN ISO/TS 82304-2 and the Label2Enable Certification Scheme**

The Technical Specification CEN ISO/TS 82304-2 (henceforth referred to as 82304-2 or the TS), published in 2021 [7], was based on 26 existing frameworks, the principles of EU-level legislation (specifically the Medical Device Regulation and General Data Protection Regulation), and the input of 20 subject-matter experts. After that, 83 experts from six continents – mostly from Europe – participated in a two-round Delphi study to come to a consensus on a framework for evaluating the quality of health apps. Experts included specialists in sub-aspects of health app quality assessment and key stakeholders from 8 groups, including medical professionals, medical organizations, and healthcare authorities. Next, 11 COVID-19 symptom apps were tested for the Dutch Ministry of Health and experiences used to refine the framework [8]. The Label2Enable project assessed with several studies the compatibility of 82304-2 and related products and services with multi-stakeholder needs and how to further increase that compatibility [9-13]. The Label2Enable 82304-2 handbook for certified app assessment organizations is informed by international scientific studies on health app quality and risks, in particular as to what is considered sufficient evidence (referred to as “pass / fails”) and assessment approach (yes/no/not applicable “sub-questions” that guide the app assessment organizations in a health app assessment).

The 82304-2 global assessment framework includes 74 requirements within 4 quality aspects, as listed in **Table S2**. Depending on for instance intended use and processing of personal data, at most 67 requirements are score-impacting. To qualify for the 82304-2 quality label, currently, 4 requirements are mandatory; however, the number of mandatory requirements will likely increase depending on the jurisdiction it is used in. Fourteen non-score-impacting requirements, in part in a separate chapter Product information, are mainly used to inform the descriptive part of its health app quality label, related more detailed health app quality report and to enable adequate assessment. Thus, in total, 82304-2 is composed of 81 requirements [7].

**Table S2.** Quality aspects and sub-aspects of the CEN ISO/TS 82304-2 assessment framework, along with the number of requirements in each aspect and sub-aspect, in parentheses [7].

| **Quality aspect** | **Sub-aspects** |
| --- | --- |
| **Healthy and safe**  ***28 requirements*** | - Health requirements (7) - Health risks (6) - Ethics (2) - Health benefit (11) - Societal benefit (2) |
| **Easy to use**  ***13 requirements*** | - Accessibility (5) - Usability (8) |
| **Secure data**  ***21 requirements*** | - Privacy (10) - Security (11) |
| **Robust build**  ***12 requirements*** | - Technical robustness (8) - Interoperability (4) |

Each requirement within 82304-2 is formulated as a question. Each question is accompanied by information to guide both assessors and manufacturers: (1) a condition defining when the requirement is applicable (if relevant), (2) its purpose, (3) the response options (yes/no including for some not applicable or choosing from a response option list, for instance), (4) the evidence required to pass the requirement, and (5) explanatory notes.

The assessment results consist of 4 scores, one for each quality aspect, which are visualized in a quality label similar to that of the EU Energy Label (**Figure S2**). Additionally, a health app quality report is generated, for which the Label2Enable health professional advisory board created guidance. This report will be made publicly available, and provides app information from the 81 requirements assessed that is considered useful for potential users, health professionals, medical societies and health systems in their decision-making on using an app, recommending an app, creating guidance on recommending apps and reimbursing and integrating an app. The 82304-2 label is issued if an app has provided all the evidence needed to enable an assessment and subsequently the assessor confirms all the mandatory requirements are passed. CEN ISO/TS 82304-2 including the full detail of its requirements can be purchased online on the International Organization for Standardization website [7].


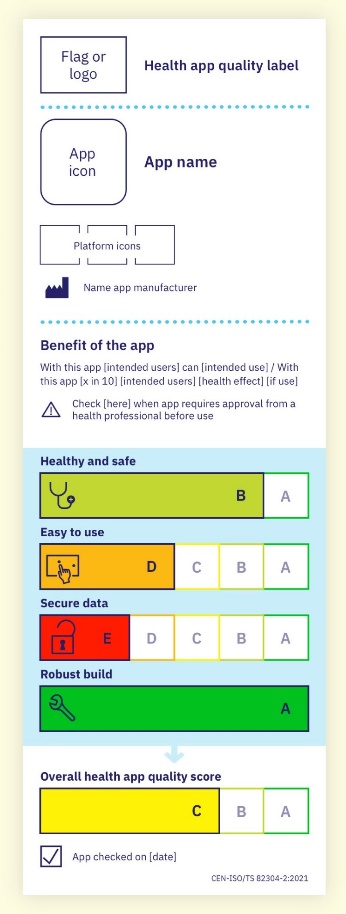


**Figure S2.** CEN ISO/TS 82304-2 quality label [14].

The EU-Funded Label2Enable Project has iteratively co-created and tested the Label2Enable handbook for app assessment (a document to guide developers and assessors in the process), which evolves the assessment framework from expert assessment to consistent self-explanatory manual assessment by adding additional guidance and evidence needed, subquestions to each requirement and pass/fail criteria. The handbook will be operational in 2025. The evolution of the handbook has been achieved by different means. First, by testing the assessment with 24 intentionally diverse apps from a wide range of medical specialities, with 5 assessment organisations from 5 different countries. Second, by performing comparative analyses with several European health app focused Health Technology Assessment (HTA) frameworks. Third, by aligning with EU level legislation and values, the needs of health professionals to confidently recommend apps as voiced in the guidance on the health app quality report, and for some of the requirements scientific findings and in more detail standardization.

In addition, the Label2Enable certification scheme, that defines the certification process, has also been developed [15]. The intent is to issue the first 82304-2 quality labels with these documents once the Label2Enable 82304-2 handbook and certification scheme are operational. Certification of the first 100 apps, multi-stakeholder evaluation including further fine-tuning of the health app quality report, and an exploration of how automated assessment could further enhance the assessment process is intended in the upcoming demonstration phase.

**References**

1. Govern. Generalitat de Catalunya. El Govern aprova el Pla de Mobilitat “mHealth.cat”, que aproparà serveis sanitaris i de benestar a través de tecnologies mòbils. 2015. Available from: https://govern.cat/salapremsa/notes-premsa/279950/govern-aprova-pla-mobilitat-healthcat-que-apropara-serveis-sanitaris-benestar-traves-tecnologies-mobils [Accessed Sep 19, 2024]
2. Fundació TIC Salut Social. Desenvolupament d’aplicacions mòbils en l’àmbit de la salut i l’atenció a la persona. 2021. Available from: https://ticsalutsocial.cat/wp-content/uploads/2021/07/DesAppsAmbitSalutIAtPersona.pdf [Accessed Sep 19, 2024]
3. López Seguí F, Pratdepàdua Bufill C, Rius Soler A, De San Pedro M, López Truño B, Aguiló Laine A, Martínez Roldán J, Garcia Cuyàs F. Prescription and Integration of Accredited Mobile Apps in Catalan Health and Social Care: Protocol for the AppSalut Site Design. JMIR Res Protoc JMIR Publications Inc.; 2018 Dec 1;7(12). PMID:30578234
4. Fundació TIC Salut Social. Criteris de certificació d’Apps de salut o benestar. 2021. Available from: https://ticsalutsocial.cat/wp-content/uploads/2021/09/2021_Criteris-certificacio_CAT_v3.0.pdf [Accessed Sep 19, 2024]
5. Fundació TIC Salut Social. Guia del procés d’acreditació per Apps de salut o benestar. 2021. Available from: https://ticsalutsocial.cat/wp-content/uploads/2021/07/guia-proces-acreditacio-fts.pdf [Accessed Sep 19, 2024]
6. Fundació TIC Salut Social. Digital Assets Directory. Available from: https://ticsalutsocial.cat/en/que-fem/digital-assets-for-citizens/assets/ [Accessed Sep 19, 2024]
7. International Organization for Standardization. ISO/TS 82304-2:2021 - Health software — Part 2: Health and wellness apps — Quality and reliability. Available from: https://www.iso.org/standard/78182.html [Accessed Sep 19, 2024]
8. Hoogendoorn P, Versluis A, van Kampen S, McCay C, et al. What Makes a Quality Health App-Developing a Global Research-Based Health App Quality Assessment Framework for CEN-ISO/TS 82304-2: Delphi Study. JMIR Form Res; 2023 Jan 23;7(1):e43905. PMID:36538379
9. Caiani EG, Kemps H, Hoogendoorn P, Asteggiano R, et al. Standardized assessment of evidence supporting the adoption of mobile health solutions: A Clinical Consensus Statement of the ESC Regulatory Affairs Committee. European Heart Journal - Digital Health Oxford University Press (OUP); 2024 Jun 4;00:1–15. DOI: 10.1093/EHJDH/ZTAE042
10. Hoogendoorn P, Shokralla, M, Wilemsen, R, Guldemond N, Villalobos-Quesada, M. Compatibility of the CEN ISO/TS 82304-2 Health App Assessment Framework with Catalan and Italian Health Authorities’ Needs: A Qualitative Interview Study. JMIR Preprints. 22/10/2024:67855. DOI: 10.2196/preprints.67855
11. Frey AL, Leigh S, Toro C, Pratdepàdua Bufill C, McCay C, Prenđa Trupec T, D'Avenio G, Kok M, Montvila A, Goedecker P, Hoogendoorn P. Preferences and willingness to pay for health app assessments among healthcare stakeholders: a discrete choice experiment. JMIR Preprints. 17/02/2024:57474 DOI: 10.2196/preprints.57474
12. Frey AL, Matei D, Phillips B, McCabe A, Fuller R, Laibarra B, Alonso L, de la Hoz V, Pratdepadua Bufill C, Llebot Casajuana B, D'Avenio G, Sottile PA, Rocchi LM, Errera M, Laaissaoui Y, Cardinal M, Kok M, Hoogendoorn P. Testing and iterative improvement of the CEN ISO/TS 82304-2 health app quality assessment: a pilot study. JMIR Formative Research. DOI: 10.2196/64565
13. Biliunaite I, van Gestel L, Hoogendoorn P, Adriaanse M. Value of a quality label and European healthcare professionals’ willingness to recommend health apps: An experimental vignette study. J Health Psychol J Health Psychol; 2024 Aug 2; PMID:39096027
14. Label2Enable. About CEN-ISO/TS 82304-2. Available from: https://label2enable.eu/about-cen-iso-ts-82304-2 [Accessed Sep 19, 2024]
15. Label2Enable. About Label2Enable. Available from: https://label2enable.eu/about-the-project [Accessed Sep 19, 2024]
