# Supplementary material for: Integrating CEN ISO/TS 82304-2 in the Catalan Health App Assessment Framework: Comparative Case Study
Source: JMIR Mhealth Uhealth. 2025 Jun 4;13:e67858. doi: 10.2196/67858 (PMC12154936; doi:10.2196/67858)
Supplement: Multimedia Appendix 2 [file mhealth-v13-e67858-s002.docx]

**Multimedia Appendix 2. Governance and Maintenance of the CEN ISO/TS 82304-2 and TIC Salut Social Foundation Assessment Frameworks**

**TIC Salut Social Foundation Assessment Framework**

The mHealth Office of TIC Salut Social Foundation (FTSS) is within the Catalan Ministry of Health. The Office is responsible for the governance of the health apps assessment framework, as established in the Mobility Plan mHealth.cat [1]. This includes the maintenance of the framework and its requirements, the validation of the assessment results for each app, and the issuance of the FTSS Certification Seal. The assessments are performed by the “Functional Experts Committee” from health and wellness societies, and by “Quality Assessors”, in their capacity of experts in their fields. These professionals can be either FTSS professionals or external parties.

The review and maintenance of the FTSS assessment framework has been performed regularly to ensure its quality, with improvements applied every two to three years. In 2019, levels of obligatoriness of some requirements were changed based on the results of the performed assessments. In 2021, the phrasing of the requirements was improved to better specify the conditions to fulfil the requirements. In 2023, the current study was initiated in order to assess the possibility of integrating CEN ISO/TS 82304-2 (“82304-2”).

To improve and approve the framework, a process of 11 steps was set as a standard for reviewing and maintaining the requirements regularly. As depicted in **Figure 2** (in the main body of the manuscript), first the FTSS mHealth Office (1) establishes the aspects and their set of requirements; and then, for the ‘Clinical contents and functionality’ aspect (2) gathers the Functional Experts Committee to perform actions 3-5. Then the Committee (3) validates this set of requirements through clinical practice, knowledge, guidelines and research papers, and defines modifications and new requirements within the domain when necessary. Next, the Committee (4) designates the assessment methodology by specifying the elements (content, functionalities and clinical evidence) that must be evaluated in the downloaded app; and (5) defines the level of obligatoriness of each requirement according to the importance of the concept and the experience acquired with the assessment of this requirement. In parallel, for the rest of aspects and set of related requirements, the mHealth office (6) consults technology and data protection experts, as well as the quality assessors that do the assessment. The experts and assessors (7) approve or adjust the requirements within their field of knowledge, by consulting Catalan legislation and national (Spanish) legislation and if relevant, common practice; (8) define the assessment methodology for each requirement (how the app needs to be used to form an opinion about it and assess the requirement); and (9) designate the level of obligatoriness of each requirement. Finally, for all aspects, the FTSS mHealth Office (10) evaluates whether reassessment of the app will be permitted after the manufacturer has been able to address failed requirements, as well as which will be the conditions to directly withdraw the label; and (11) approves the overall assessment framework by ensuring the consistency of the set of requirements, the assessment process and the results obtained for the assessed apps.

**CEN ISO/TS 82304-2 and Label2Enable Certification Scheme**

Label2Enable has assigned a Scheme Owner, Stakeholders and Expert Organisation, which also includes representatives of the Conformity Assessment Bodies and the Certification Bodies, to jointly ensure the maintenance and implementation of the Label2Enable certification scheme and related Label2Enable 82304-2 handbook for certified app assessment organizations [2]. The Label2Enable 82304-2 handbook is maintained by the Stakeholders and Expert Organization annually or more frequently if needed. In addition, to attain trust and impact of the 82304-2 quality label and report, a methodology for guaranteeing a proper definition and maintenance of each 82304-2 requirement has been defined. The methodology is centred on each requirement rather than on aspects and involves 9 steps. It has been represented in a colorful scheme (see **Figure 3** in the main body of the manuscript).

Thus, (1) for each 82304-2 requirement, (2) subject-matter experts are recruited and consulted. They help operationalize each requirement, considering (3) the rationale of the assessment, ranging from EU level legislation and values, standardization, key stakeholder needs, common practice and research findings. The rationale informs (4) the method of assessment where an effort was made to evolve from expert assessment to manual assessment and going forward using trusted existing assessments and automated tooling. These assessment methods determine (5) the skills assessors need and thus their training requirements. The rationale also determines (6) the rigor of the assessment which is set with “pass/fail” definitions, referring to what is sufficient evidence. Next, (7) the conditions for reassessment are decided, (8) the robustness is evaluated, (9) and key stakeholders and authorities are consulted to ensure the assessment is useful and proportional, which then together with changes in the rationale, such as legislation or research findings, emergence of assessment methods, and day-to-day operational experiences informs maintenance of the handbook and ultimately the revision of 82304-2.

**References**

1. Govern. Generalitat de Catalunya. El Govern aprova el Pla de Mobilitat “mHealth.cat”, que aproparà serveis sanitaris i de benestar a través de tecnologies mòbils. 2015. Available from: https://govern.cat/salapremsa/notes-premsa/279950/govern-aprova-pla-mobilitat-healthcat-que-apropara-serveis-sanitaris-benestar-traves-tecnologies-mobils [Accessed Sep 19, 2024]
2. Label2Enable. Results. Available from: https://label2enable.eu/results [Accessed Sep 19, 2024]
