# Supplementary material for: Integrating CEN ISO/TS 82304-2 in the Catalan Health App Assessment Framework: Comparative Case Study
Source: JMIR Mhealth Uhealth. 2025 Jun 4;13:e67858. doi: 10.2196/67858 (PMC12154936; doi:10.2196/67858)
Supplement: Multimedia Appendix 3 [file mhealth-v13-e67858-s003.docx]

**Multimedia Appendix 3. Certification Workflows of the CEN ISO/TS 82304-2 and TIC Salut Social Foundation Assessment Frameworks**

**TIC Salut Social Foundation Certification Process**

The TIC Salut Social Foundation (FTSS) certification process comprises 7 stages [1], as depicted in **Figure 4** of the main body of the manuscript:

1. Perform self-evaluation test *(optional)*: On the FTSS website, manufacturers can carry out a self-evaluation test [2], which includes 31 yes/no questions to assess the app’s maturity and readiness to undergo the assessment. The questions consist of a selection of the most important requirements of the FTSS assessment framework.
2. Express interest in certification: Manufacturers must contact the FTSS mHealth Office to express their interest to certify their app. Then, FTSS sends them a submission form with 52 fields, which they must answer in writing and submit to start the assessment process. This form requires basic information about the app (app owner and manufacturer, contact details, app product information, as well as the app icon and screenshots) and information specifically gathered for the app’s assessment (app functionality and utility, intended use, sensitive data usage, impact, permissions required, professionals involved, etc.).
3. Sign assessment agreement: Both parties sign a written agreement to engage in the assessment, which establishes the obligations of each.
4. Test app and classify it into risk level: The quality assessors perform an initial validation to ensure that the app is sufficiently mature to undergo the assessment and that it functions according to the description provided in the form supplied by FTSS. The app is then classified, i.e. assigned a risk level between 1 and 3, with 3 being the maximum risk. This classification is based on intended use, sensitive data usage and population impact (percentage of intended users among the total population of Catalonia) and determines the level of obligatoriness of each requirement.
5. (Re-)Assess requirements through the app: The 120 requirements are evaluated by 5 quality assessors guided by an assessment document provided by FTSS. The document contains, along with the requirement sentence and description, a text field where the assessor can register the assessment result (Yes/No/Not applicable). A text field for additional comments is provided to justify why the requirement was not fulfilled, along with app screenshots if necessary. This document is sent as a final report to the manufacturer.

- “Clinical contents and functionality” quality aspect: The associated requirements are evaluated by 3 independent assessors from the Functional Experts Committee (which possess general healthcare knowledge but are not experts in the specific health issue of the app). They use the information provided in the initial form, download the app and analyse its content and functionalities to decide whether it passes each of the requirements for this aspect. They discuss their findings to achieve consensus and, if necessary, investigate clinical practice guidelines and papers for the health or wellness condition to which the app is directed.
- Remaining quality aspects: The other requirements are assessed by 1 technology expert and 1 accessibility expert from the Quality Assessors. For this purpose, the app is downloaded, used and analysed, with the help of the information provided by the manufacturer in the initial form. Additionally, Quality Assessors perform tests with different devices to ensure compatibility of the published app versions.

1. Issue (or withdraw) report and certificate: The assessment results are compiled in a final report, along with the numerical score obtained based on the successfully passed requirements. If all mandatory requirements are met, the FTSS Certification Seal is granted to the specific app version assessed. The agreement (see stage 3) states that manufacturers have the obligation to notify FTSS in case of major changes to the app that could affect the assessment results. In turn, FTSS monitors annually the versions of the app available in marketplaces to ensure compliance. If major changes were to be detected by FTSS, the Seal could be withdrawn.
2. Publish (or not) into FTSS Apps Directory: Certified apps are published and highlighted in the FTSS Apps Directory [3] with very similar information to the one provided in app stores, but highlighting healthcare characteristics. FTSS annually monitors changes of apps in marketplaces to update the information on the Directory.

This process can be iterative, meaning that once the final report is provided to the manufacturers, they can decide to make changes to the app within the next 6 months and request a re-assessment of specific requirements (stage 5). If the changes take more time to be applied and/or the app is changed significantly, it is necessary to renew the agreement (stage 3) [1].

**Label2Enable Certification Scheme and Label2Enable 82304-2 Handbook**

The documentation of the Label2Enable certification scheme defines its implementation and related bodies involved: the Scheme Owner, Stakeholders and Expert Organisation, Certification Bodies, Conformity Assessment Bodies and the Client (manufacturer).

In addition, Label2Enable has designated a more extensive prescriptive document for the assessment, the Label2Enable 82304-2 handbook for certified app assessment organizations. This handbook includes, for each 82304-2 requirement, the information contained in the CEN ISO/TS 82304-2 Technical Specification (“TS’ or “82304-2”), additional guidance (notes) and evidence needed from manufacturers, sub-questions (sub-requirements), the “pass/fail” definitions (what is sufficient evidence) and what are mandatory requirements given EU-level legislation and values [4].

Overall, the certification of each app is defined as a 7-stage process, which is also summarized in **Figure 4**.

1. Express interest in certification: A manufacturer can contact one of the Label2Enable certified Conformity Assessment Bodies to express interest in certification.
2. Sign assessment agreement: Both parties sign the certification agreement which specifies the terms and conditions (eg, tasks and responsibilities of both parties, rules of validity, renewal and withdrawal of the certification). The manufacturer is given access to the handbook and further guidance available (eg, a tutorial).
3. Supply (or update) responses and evidence: To enable app assessment, manufacturers must fill out a questionnaire for all applicable 82304-2 requirements. Applicability and thus the number of requirements is dependent on intended use, existence of health information in the app, existence of health and societal benefit, compliance with accessibility guidelines, processing of personal data, and existence of interoperability. Aside from the questionnaire, the manufacturer must supply evidence for each of the requirements, following the additional guidance detailed in the handbook. They must also provide the assessors (Conformity Assessment Body) access to the app in full, including if applicable any required accessories.
4. (Re-)Assess requirements through evidence and app: The assessors use the evidence provided by the manufacturers and access to the app to evaluate for each of the sub-requirements and subsequently requirements if the evidence is sufficient (“pass”) or insufficient (“fail”). The handbook supplies a predefined algorithm, the pass / fail definition, which details which of the sub-requirements must be met to pass the requirement as a whole. In case of uncertainties as to the responses or evidence provided, or difficulties to access or use the app and potential accessories, the manufacturer representative is contacted.
5. Share findings, allow fixes: Once all requirements have been assessed, the health app quality scores for each quality aspect and the overall score are calculated. If the app has passed all mandatory requirements, the health app quality label and health app quality report can be generated and issued. The report includes the assessment results (pass / fail) and factual information about the app that aims to avoid multi-stakeholder duplication of assessment efforts. The results are shared with the manufacturer, enabling within a given timeframe a possibility to upgrade (fix issues in) the app and resubmit evidence to enable (re)assessment.
6. Issue and publish (or withdraw) label and report: The Certification Body issues the health app quality label and report and certificate of conformity. A verification service enables stakeholders to establish if a label is indeed accurate. If conditions are not met the label can be suspended and withdrawn.
7. Do surveillance or apply for reassessment: The Stakeholders and Expert Organisation define the requirements for surveillance activities, to provide assurance that the certified products continue to fulfil the specified requirements described.

**References**

1. Fundació TIC Salut Social. Guia del procés d’acreditació per Apps de salut o benestar. 2021. Available from: https://ticsalutsocial.cat/wp-content/uploads/2021/07/guia-proces-acreditacio-fts.pdf [Accessed Sep 19, 2024]
2. Fundació TIC Salut Social. Self-assessment test. Available from: https://ticsalutsocial.cat/en/que-fem/digital-assets-for-citizens/test-dautoavaluacio/ [Accessed Sep 19, 2024]
3. Fundació TIC Salut Social. Digital Assets Directory. Available from: https://ticsalutsocial.cat/en/que-fem/digital-assets-for-citizens/assets/ [Accessed Sep 19, 2024]
4. Label2Enable. Results. Available from: https://label2enable.eu/results [Accessed Sep 19, 2024]
